# Successful Right Ventricular Tachycardia Ablation in a Patient with Left Ventricular Non-compaction Cardiomyopathy

**DOI:** 10.1016/s0972-6292(16)30671-4

**Published:** 2013-09-01

**Authors:** Shohreh Honarbakhsh, Irina Suman-Horduna, Lilian Mantziari, Sabine Ernst

**Affiliations:** 1 Royal Brompton and Harefield NHS Foundation Trust, United Kingdom; 2 NIHR Cardiovascular Biomedical Research Unit, Royal Brompton and National Heart and Lung Institute, Imperial College London

**Keywords:** left ventricular non-compaction, ventricular tachycardia, electrophysiology study, ablation

## Abstract

We report a case of a 67-year old male with a recent diagnosis of left ventricular non-compaction (LVNC), initially presenting with symptomatic ventricular ectopy and runs of non-sustained ventricular tachycardia (VT). This ventricular arrhythmia originated in a structurally normal right ventricle (RV) and was successfully localized and ablated with the aid of the three-dimensional mapping and remote magnetic navigation.

## Case presentation

A 67-year old Caucasian man with a recent diagnosis of left ventricular non-compaction (LVNC) and previous ablation of ventricular extrasystoles (VEs) in the right ventricular outflow tract was transferred as an emergency to our institution for electrophysiological study and further management of multiple episodes of non-sustained VT. He had a two-year history of palpitations on exertion with associated pre-syncopal symptoms and one syncopal episode.

**Figure 1:**
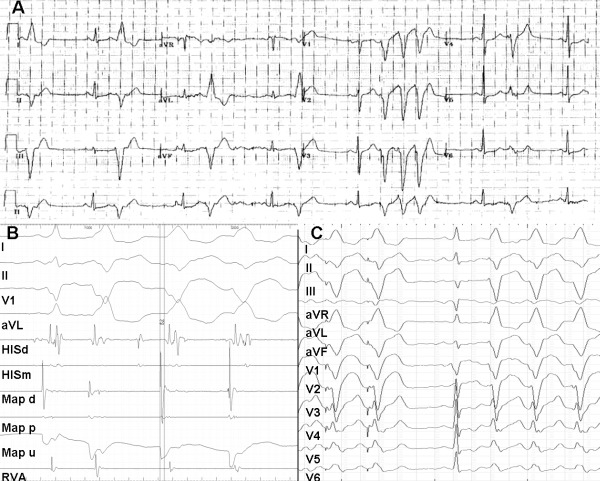
A. Resting 12 lead ECG demonstrating ventricular bigeminy and ventricular salvos. B. Local bipolar (-28msec) and unipolar signal before ablation (speed 100mm/s). C. Pace map at the ablation point. HISd - His distal; HISm - His middle; Map d - Map distal; Map p - Map proximal; Map u- Map unipolar; RVA - Right ventricular apex.

The 12-lead ECG on admission showed ventricular bigeminy and telemetry revealed multiple short runs of monomorphic non-sustained ventricular tachycardia (VT) of similar morphology to the VEs, and without haemodynamic compromise (Figure 1A). The QRS morphology of the ectopic beats was of left bundle branch block morphology, transition by V3/V4, superior axis and positive deflection in leads I and aVL, suggestive of a basal right ventricular origin. Transthoracic echocardiogram showed global hypokinesis of the LV with moderately reduced ejection fraction of 44% and prominent trabeculation of the lateral and apical walls. The RV was structurally normal.

## Invasive electrophysiological study

**Figure 2:**
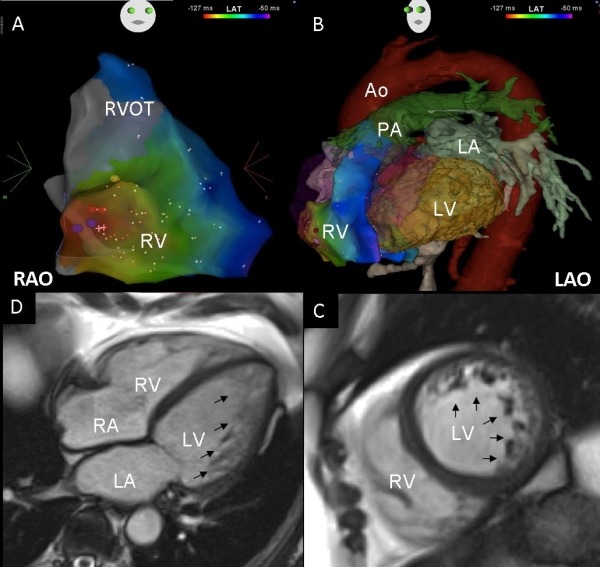
A and B. A right anterior oblique view of the right ventricular activation map during ventricular ectopy (A) and a left anterior oblique view of the activation map merged with a 3D reconstruction of a cardiac magnetic resonance scan (B) illustrate the earliest activation basally, adjacent to the tricuspid annulus at the 8-9 o'clock position (red colour). C and D. Cardiovascular magnetic resonance steady-state free precession mid-ventricular short axis slice (C) and apical four-chamber view (D), showing pronounced trabeculations (arrowed), predominantly involving the lateral wall and the apex, with a thinner compacted layer of myocardium. RV - Right ventricle; RVOT - Right ventricular outflow tract; RA - Right atrium; LA - Left atrium; LV - Left ventricle; PA - Pulmonary artery; Ao - Aorta; RAO - Right anterior oblique; LAO - Left anterior oblique

At the beginning of the electrophysiological study the patient was in sinus rhythm with monomorphic ventricular bigeminy. A three dimensional map of the RV was acquired using magnetic navigation in conjunction with CARTO 3 RMT (Biosense Webster, Brussels, Belgium) (Figure 2A and 2B). Activation map of the VE depicted the earliest area (-28msec) at the base of the RV towards the infero-lateral aspect of the tricuspid annulus at the 8 o'clock position (Figure 1B), and was suggestive of a focal mechanism. At this earliest site, a 12/12 pace map (Figure 1C) was obtained and subsequent ablation led to warming-up with repetitive non-sustained VT with the same morphology as the VEs, followed by termination. During a waiting time of 30 min, no further VEs or VT were inducible by programmed RV stimulation or occurred spontaneously at baseline or during provocation with intravenous Isoprenaline.

Continuous monitoring for 48 hours post-procedure showed no further VEs /VT off any anti-arrhythmic medication.

## Cardiac magnetic resonance findings

In order to identify if there was any underlying anatomical substrate for the right ventricular origin of the arrhythmia in this case, we reviewed the images from a pre-ablation cardiac magnetic resonance (CMR) scan. The RV free wall at the site of the origin of the arrhythmia close to the tricuspid annulus (as determined from the CARTO merge 2D slices) was carefully reviewed in all views. The appearance of the LV confirmed non-compaction according to the CMR criteria described by Petersen [1] (Figure 2C and 2D), with a ratio between non-compacted and compacted myocardium of >3. A cut-off value of >2.3 for the ratio of non-compacted to compacted myocardium at end-diastole was shown to provide a sensitivity, specificity, and positive and negative predictive values of 86%, 99%, 75%, and 99%, respectively [1]. The appearance of the RV free wall was normal with no discernible pathology in the targeted area on steady-state free precession cines or late gadolinium images.

## Discussion

In this patient with LVNC and VT, the electrophysiological study demonstrated that the VT originated from a structurally normal RV. LVNC is a rare congenital cardiomyopathy with a reported incidence of 0.05% in adults [2]. It can occur in isolation or affect both ventricles [2]. It is believed to be secondary to the arrest of normal embryogenesis of the endocardium and myocardium resulting in the failure of trabecular compaction of the developing myocardium [3,4]. Diagnosis is predominantly made through identifying established echocardiographic criteria such as two-layer arrangement consisting of a thin epicardial band and a much thicker non-compacted endocardial layer [3]. Patients may be asymptomatic, present with congestive heart failure, ventricular arrhythmias or systemic emboli [2,4]. An association with WPW syndrome [4] has also been reported in this cohort.

With an abnormal LV, it is expected that the substrate for arrhythmia is located in the LV as a result of abnormal trabeculations creating a substrate for left VT. However, in our patient with LVNC the VT originated from a structurally normal RV. Interestingly, the CMR showed a normal RV and this is regarded as a highly effective imaging modality to distinguish the compacted and non-compacted myocardial layers [1]. However, normal RV trabeculations can be difficult to differentiate from those seen in non-compaction cases with biventricular disease. Therefore, it is possible that in this particular case the RV had un-identified abnormal trabeculations acting as a potential trigger for the arrhythmia.

Another hypothesis could be that the VT originated from the ventricular end of an accessory pathway that has regressed, particularly in view of the fact that the origin of arrhythmia was close to the tricuspid annulus. WPW syndrome has been reported in these patients [4]. However, although ventricular automaticity arising from a ventricular end of a Mahaim accessory pathway has been previously described [5], none of the ECGs in this case showed features of pre-excitation.

Even though it is possible that the occurrence of these findings is co-incidental, this case emphasises that patients with LVNC have an arrhythmogenic substrate that can extend beyond the LV. This could be secondary to either structural abnormalities that are below the resolution of the currently available imaging modalities or can be the result of microscopic changes such as altered ion channels or signalling pathways.
